# Effect of clinical levels of misonidazole on the response of tumour and normal tissues in the mouse to alkylating agents.

**DOI:** 10.1038/bjc.1982.111

**Published:** 1982-05

**Authors:** J. M. Brown, D. G. Hirst

## Abstract

Experiments were carried out to determine whether the enhancement of alkylating-agent cytotoxicity seen after large single doses of misonidazole (MISO) in mouse tumours can also be achieved by prolonged exposure to low MISO levels similar to those which can be tolerated clinically. The level in mouse blood plasma could be maintained at about 100 micrograms/ml for 7 h by injecting small doses of MISO every 1/2 h. The effect of this treatment in combination with cyclophosphamide (CY) or melphalan (L-PAM) was studied in the RIF-1 tumour, using regrowth delay and cell-survival cloning assays. In each case, prolonged exposure to low levels of MISO gave enhancement ratios very close to those obtained with a large single dose. ERs of 1.6-2.0 were obtained with CY and 1.8-2.2 with L-PAM over the range of alkylating-agent doses used. In experiments with CY the response of 2 normal-tissue systems, marrow and WBC count, was also studied. No significant enhancement of CY damage occurred in either case. In the L-PAM experiments the LD50/30 and WBC counts were determined as normal-tissue end points. Multiple MISO had no effect. Our results show that levels of MISO which can be achieved safely in man yield good enhancement of the tumour cytotoxicity of 2 widely used chemotherapeutic agents without increasing the damage to normal tissues.


*Br. J. Cancer* (1982) **45**, 700

### EFFECT OF CLINICAL LEVELS OF MISONIDAZOLE ON THE RESPONSE OF TUMOUR AND NORMAL TISSUES IN THE MOUSE TO ALKYLATING AGENTS

J. M. BROWN* AND D. G. HIRST

*From the Department of Radiology, Stanford University School of Medicine, Stanford, California* 94305, *U.S.A.*

Received 17 September 1981 Accepted 26 January 1982

**Summary.—Experiments were carried out to determine whether the enhancement of alkylating-agent cytotoxicity seen after large single doses of misonidazole (MISO) in mouse tumours can also be achieved by prolonged exposure to low MISO levels similar to those which can be tolerated clinically.**

**The level in mouse blood plasma could be maintained at about 100 $\mu$g/ml for 7 h by injecting small doses of MISO every 1/2 h. The effect of this treatment in combination with cyclophosphamide (CY) or melphalan (L-PAM) was studied in the RIF-1 tumour, using regrowth delay and cell-survival cloning assays. In each case, prolonged exposure to low levels of MISO gave enhancement ratios very close to those obtained with a large single dose. ERs of 1·6–2·0 were obtained with CY and 1·8–2·2 with L-PAM over the range of alkylating-agent doses used.**

**In experiments with CY the response of 2 normal-tissue systems, marrow and WBC count, was also studied. No significant enhancement of CY damage occurred in either case. In the L-PAM experiments the $LD_{50/30}$ and WBC counts were determined as normal-tissue end points. Multiple MISO had no effect.**

**Our results show that levels of MISO which can be achieved safely in man yield good enhancement of the tumour cytotoxicity of 2 widely used chemotherapeutic agents without increasing the damage to normal tissues.**

ELECTRON-AFFINIC AGENTS, such as the 2-nitroimidazole misonidazole [1-(2-nitroimidazole-l-yl)-3-methoxypropan-2-ol, Ro-07-0582, MISO], have been shown to be effective sensitizers of the cytotoxic effects of radiation on hypoxic cells *in vitro* and in tumours *in vivo* (Adams, 1977) and clinical trials of MISO in combination with radiation therapy are in progress in many centres.

Recently it has been shown that MISO is capable of sensitizing tumours in mice to a variety of alkylating agents including cyclophosphamide, melphalan and the nitrosoureas (Rose *et al.*, 1980; Clement *et al.*, 1980; Tannock, 1980; Martin *et al.*, 1981; Law *et al.*, 1981; Twentyman, 1981; Siemann, 1981; Mulcahy *et al.*, 1981). Although most of these authors have found that the cytotoxicity of the alkylating agents to the *normal* tissues is enhanced by MISO, almost all have concluded that the enhancement of the tumour response is greater than that of the normal tissues, producing a therapeutic gain. Tannock (1980), however, found no therapeutic advantage for the combined treatment of MISO with cyclophosphamide (CY) or BCNU, but Law *et al.* (1981) have suggested that this is only true at high doses of the alkylating agents, and that at lower doses there is

* To whom correspondence and reprint requests should be sent.

greater sensitization of the tumour cells than of the cells of the normal tissue. These authors show further that the therapeutic gain at moderate to low doses of CY could be exploited by multiple fractions of the combined treatments.

A major question not resolved by any of the above studies is whether the differential enhancement of the cytotoxicity of alkylating agents to tumour cells would occur at clinically realistic dose-levels of MISO. Most authors for example have used MISO doses of 600–1000 mg/kg, which produce plasma levels of MISO 5–10× those that can be achieved in humans. In those studies in which lower doses of MISO have been used, the results have been equivocal. Twentyman (1981) found that the sensitization of the RIF-1 and KHT tumours to CY was considerably reduced at 300 mg/kg MISO, and although in our own studies with the RIF-1 rumour we found significant sensitization of the cytotoxicity of CY at MISO doses of 125 and 250 mg/kg, the effect was less than that seen at higher doses of MISO. In his study with the KHT sarcoma, Siemann (1981) found a dose-modification factor for CCNU toxicity of 1·9 with a MISO dose of 250 mg/kg, but lower doses were not tested. Thus it is far from clear whether any useful enhancement of the cytotoxicity of alkylating agents on tumours will occur at the maximum plasma concentrations of MISO that can be achieved in humans (~150 $\mu$g/ml). On the other hand, the plasma half-life of MISO in the mouse is about one tenth that in man, and it is therefore possible that prolonged exposure of tumour cells to MISO, even at relatively low doses, might increase its sensitizing effect. In the present experiments we have attempted to answer this question by simulating in the mouse the prolonged low levels of MISO which can be tolerated in humans, in order to determine whether useful sensitization of the cytotoxicity of CY on tumour cells might be achieved by MISO in the clinic.

### MATERIALS AND METHODS

### *Tumour studies*

The RIF-1 tumour used in the present study is a nonimmunogenic sarcoma in its syngeneic host, the C3H/Km mouse, and has been developed for *in vivo* and *in vitro* assay (Twentyman *et al.*, 1980). Solid tumours were used in 3–4 month old female C3H/Km mice by inoculating $2 \times 10^5$ cells in a volume of 0·05 ml, either into the base of the gastrocnemius muscle or intradermally in the flank. Growth of the leg-implanted tumours was followed by measuring 2 leg diameters at right angles, and tumour volume was estimated from a calibration curve for tumour weight plotted as a function of the product of the 2 leg diameters (Twentyman *et al.*, 1979). Drug treatments were given when the tumours were 300–600 mg. The volume of the flank tumours was followed by measuring 3 mutually perpendicular diameters of the tumour and using the formula:

$$\pi/6 \times d_1 \times d_2 \times d_3$$

The response to drug treatments was investigated by 2 methods. For the regrowth-delay assay, 10 mice were included in each experiment group and tumours were measured at 3× per week. To compare treatments, the number of days required to reach 4× the mean treatment volume was determined from growth curves plotted for each animal. Geometric means and standard errors were calculated for each treatment group.

For the cell-survival assay, tumours were excised 24 h after treatment. Two to 4 tumours were pooled and a single-cell suspension was prepared by mincing the tissue and incubating it with an enzyme cocktail of 0·02% collagenase, 0·02% DNAse and 0·05% pronase. The number of cells excluding trypan blue was counted using a haemacytometer (cell yield ~$2 \times 10^8$ cells/g) and appropriate dilutions were plated into polystyrene Petri dishes containing complete Waymouth's medium with 10% foetal calf serum. Colonies of at least 50 cells were counted after 13 days' incubation at 37°C. The plating efficiency (PE) for control tumours was ~30%. Surviving fractions were calculated using either PE or number of clonogenic cells/g tumour. There was no significant difference between the results obtained using the 2 methods, so only surviving fractions estimated from PE are shown in the figures.

*Normal-tissue studies*

*Marrow stem cells.*—The effect of the drug treatments on the survival of marrow stem cells was studied using the spleen-colony assay (Till & McCulloch, 1961). Marrow cells flushed from the femurs of treated C3H/Km mice (usually 4 mice per group) were injected into the tail veins of preirradiated recipients. Two dilutions of marrow cells were always used, with 6 mice per dilution. The number of colonies counted on the spleens of these animals after 7–8 days was used to determine the survival of injected marrow cells.

*White-cell counts.*—Previous experiments have shown that the number of WBC in the peripheral blood declines for several days after treatment with CY (Law *et al.*, 1981), reaching a minimum at about 4 days before recovery begins. In the present experiments 5$\mu$l blood samples were taken from the tails of mice (6 per group) 4 days after MISO and CY treatments. The blood was diluted in 95 $\mu$l of 2% glacial acetic acid to lyse the red blood cells and counts of total leucocytes were made. Results were expressed as the number of cells/mm$^3$ in the original blood sample.

*$LD_{50/30}$.*—Normal-tissue toxicity in the L-PAM study was assessed by determining the number of animals dying within 30 days after doses of 6·2–14 mg/kg L-PAM, with or without multiple MISO. All the animals bore the RIF-1 tumour in the leg. At the lower doses of L-PAM animals had to be killed before 30 days because their tumours became large. Since none of the animals showed any sign of sickness at these doses, they were considered to be survivors for analysis of the data. $LD_{50}$ values and 95% confidence limits were determined by logit analysis. A similar experiment with non-tumour-bearing mice was also performed. 10 mice per dose group were used.

*Monitoring MISO levels.*—Plasma (or whole blood) levels of MISO were determined using high-performance liquid chromatography (HPLC) as follows. At regular intervals during the MISO administrations, 10$\mu$l blood samples were collected from the tails of the experimental mice in Microcap pipettes (Drummond Scientific Co.), mixed with 40 $\mu$l distilled water and stored at $-20$°C. They were subsequently analysed for MISO and desmethyl MISO levels as described previously (Workman *et al.*, 1978).

*Drug treatments*

MISO and CY were dissolved in physiological saline, but L-PAM had first to be dissolved in ethanol and further diluted in saline immediately before injection because of its instability in aqueous solution. All the drugs were injected i.p.

When given as a large single dose of 750 mg/kg, MISO was made up at a dilution of 25 mg/ml and injected at 0·03 ml/g body wt 30 min before CY or L-PAM injection. For the multiple-injection schedule, 2 dilutions of MISO were made for each experiment. In combination with CY, a high priming dose of 0·6 mmol/kg MISO was first given by injecting 0·01 ml/g body wt of a 12mg/ml solution. This was followed at 1/2 h intervals by 14 doses of 0·15 mmol/kg, by injecting 0·01 ml/g of a 3 mg/ml solution. In the L-PAM experiments, MISO doses were reduced in an attempt to match the human drug profile more closely. The priming dose was reduced to 0·24 mmol/kg and the subsequent doses reduced to 0·12 mmol/kg. The single CY or L-PAM injections were given 4 h after the first priming dose of MISO, and immediately before the 9th MISO injection. The time was chosen because in a preliminary experiment we had found that 4 h gave the maximum response, with no additional sensitization at longer times.

### RESULTS

*Cyclophosphamide*

Fig. 1 shows the response of the RIF-1 sarcoma (implanted i.m.) to various doses of CY. The mice were injected with saline (controls) or given repeated injections of MISO every 30 min. For the latter group the CY injection was given 4 h after the start of the prolonged MISO exposure, which was then continued for a further 3 h. The lower panel of Fig. 1 shows the plasma concentration of MISO in the repeatedly injected group. It can be seen that it closely simulates the levels found in humans by Urtasun *et al.* (1977) following a single oral dose of 7 g of MISO. Also shown in the upper panel of Fig. 1 are the data in the same experiment for the response of the tumours to CY given 30 min after a single, large dose of MISO (750 mg/kg).

FIG. 1.—The effect of prior treatment with saline (○, □), a large single dose of 750 mg/kg MISO (▲) or prolonged exposure (15 fractions) to low-level MISO (●, ■) on the survival of RIF-1 tumour cells exposed to a range of CY doses. The circles and squares indicate two independent experiments. Lines were drawn by eye through the saline and the multiple-MISO points (upper panel). Also shown (lower panel) are the plasma levels achieved with multiple MISO injections in one of these experiments (●). The dashed line represents a typical human exposure after the oral administration of 7 g MISO (Urtasun *et al.*, 1977).

FIG. 2.—The effect of saline (○) or prolonged MISO exposure (●) on the CY dose–response curve for marrow stem cells (CFU-S). Line was drawn through the saline + CY data.

FIG. 3.—The effect of saline (○) or prolonged low-level MISO (●) on regrowth delay of the RIF-1 tumour after various doses of CY. Points show geometric means ± s.e. Tumours grown i.m. in the leg.

Fig. 2 shows the response of marrow stem cells (CFU-S) to various doses of CY with or without prolonged MISO administration; animals were killed 24 h after CY injection. The data points at CY doses of up to 100 mg/kg were obtained from the mice used for the tumour response in Fig. 1. The higher-dose groups were obtained in a separate experiment using an identical injection schedule of MISO.

Figs 3 and 4 show dose–response curves for regrowth delay of the RIF-1 tumour in mice given various single doses of CY. The groups given MISO were subject to exactly the same repeated injection protocol of MISO every 30 min, and were injected with CY 4 h after the first MISO

FIG. 4.—The effect of the same saline +CY (○) and MISO+CY (●) treatments as in Fig. 3 on regrowth delay of the RIF-1 tumour growing intradermally in the flank.

FIG. 5.—The number of white blood cells in the peripheral blood 4 days after treatments with saline+CY (○, △) or prolonged MIS exposure+CY (●, ▲). Error bars are ±s.e. Circles and triangles represent separate experiments.

injection. The last MISO injection was given 3 h after the CY injection. Blood levels in both experiments were monitored as before, and found to be similar to those shown in Fig. 1.

One of the purposes of these two experiments was to examine the influence of the different types of hypoxia in RIF-1 tumours implanted i.m. and i.d. The radiation response of i.m. tumours indicates that they contain 25–100% of cells at an intermediate level of oxygenation, whereas intradermally (i.d.) implanted RIF-1 tumours have a radiation response which shows that they have few (~1%) fully (radiobiologically) hypoxic cells (Brown *et al.*, 1980). However, it can be seen from a comparison of Figs 3 and 4 that both i.m. and i.d. implanted tumours were sensitized about equally to the cytotoxic action of CY.

Fig. 5 shows results from two separate experiments in which WBC counts were made at the nadir of the response after CY injection (4 days). One set of data was obtained on the same mice that were used for the tumour response in Fig. 3. As with the CFU-S assay (Fig. 2), it is apparent that MISO, under the given conditions, had no cytotoxicity by itself and no sensitization to CY.

### *Melphalan*

The regrowth-delay assay was used to study the effect of multiple MISO injections on the cytotoxicity of melphalan (L-PAM). The MISO doses chosen were lower than those in the CY experiment, in an attempt to match the human levels more closely at early times after drug administration. This was successful (Fig. 6 lower panel). L-PAM treatments produced considerable tumour regression, with or without multiple MISO, but when the tumours finally regrew, their growth rate was the same as untreated tumours, showing no "tumour bed effect". These growth curves allowed accurate determinations of the time each group of tumours took to reach 4 × their volume at the time of treatment. The mean values for each treatment group are shown in Fig. 6 (upper panels). The mice received multiple injections of saline or MISO every 30 min, in combination with various doses of L-PAM. In both experiments multiple MISO was found to be dose-modifying, giving an enhancement

FIG. 6.—The upper panels show the effect in two separate experiments of different doses of L-PAM, with (●) or without (○) prolonged low-level MISO exposure, on the time to reach 4 × treatment volume. Also shown is the enhancement of L-PAM cytoxicity by a large single dose of MISO (750 mg/kg) 30 min before L-PAM injection (▲). Error bars ± s.e. The plasma levels of MISO in one of these low-level experiments are shown in the lower panel in which, the dashed line represents a typical human drug profile after a single 7g oral administration of MISO (Urtasun *et al.*, 1977).

ratio of ~2. The effect of a large single dose of MISO (750 mg/kg) is also shown for comparison. The MISO levels achieved with multiple injections in these experiments are shown in Fig. 6 (lower panel).

The response of a normal tissue to multiple MISO and L-PAM was determined using WBC counts. Fig. 7 shows the effect of multiple MISO injections. In 2 experiments no significant enhancement was apparent. The dose of L-PAM required to kill 50% of mice bearing the RIF-1 tumour in the leg was not affected by the addition of multiple MISO (ER = 1·0). An $LD_{50}$ of 11·5 ± 1·3 mg/kg (95% confidence limits) was obtained in each case. The experiment was repeated with non-tumour bearing mice with the same result.

### DISCUSSION

The principal shortcoming of nitro-imidazole radiosensitizers in radiation therapy is that while radiosensitization depends mainly on the drug concentration in the tumour at the moment of irradiation (Adams *et al.*, 1975) the dose-limiting neurotoxicity shows a dependence on the total tissue exposure to the drug. Conroy *et al.* (1979) have shown that the total exposure dose of MISO to produce

FIG. 7.—The number of white blood cells in the peripheral blood 4 days after treatments with saline and L-PAM (○, △) or prolonged MIS exposure and L-PAM (●, ▲). Data from 2 experiments. Error bars ±s.e.

neuropathy is similar in mouse and man, at about 60 mM. h. The mouse can tolerate a much higher administered dose because of a shorter MISO half-life. An obvious consequence of this is that much lower drug doses must be used in the clinic than in small experimental animals, with the expectation of a lower enhancement ratio.

The interaction between MISO and alkylating agents in tumours appears to show a very different dependence on MISO exposure. While the duration of the MISO level is not a major factor in determining radiosensitization, it is one of the main determinants of chemosensitization. The effectiveness of prolonged exposure to low levels of MISO (Figs 1, 3, 4 & 6) suggests that both the duration of MISO exposure before CY treatment and peak MISO concentration are important in determining the chemosensitization, at least over the range studied. Our results show that the prolonged exposure to low levels of MISO, close to those which are now used clinically with minimal complications, gives large ERs when used with CY or L-PAM.

The absence of enhanced toxicity in the normal tissues studied is particularly encouraging and intriguing. The reason for this lack of enhancement is unclear, but is does appear that low MISO levels do not enhance toxicity in normal tissues. Additional evidence for this is provided by experiments with mouse testis (Hirst *et al.*, unpublished). Doses of MISO below 300 mg/kg gave no enhancement of CY toxicity, whereas enhancement at higher doses was considerable.

While our highest doses of alkylating agents are higher than can be tolerated in man on a mg/kg basis, good enhancement of tumour response can be achieved at doses which are well tolerated. For example, 50 mg/kg CY in a single dose will not normally lead to severe haematological impairment, but at that dose, MISO pretreatment would be expected to yield a therapeutic gain. A more appropriate basis for comparison between mouse and human dosage of alkylating agents may be g/m$^2$, in which case the best therapeutic gain should be obtained at doses below the maximum clinically achievable. In this case MISO might be more effective in combination with several smaller doses of alkylating agents.

The dependence of enhancement on both MISO concentration and duration of exposure might suggest a mechanism related to MISO cytotoxicity to hypoxic cells. It has been shown (Moore *et al.*, 1976) that both contact time and drug concentration influence the hypoxic cell cytotoxicity of MISO *in vitro*. Conroy *et al.* (1980) showed that prolonged exposure *in vivo* to MISO alone at levels similar to those used in the present study reduced the surviving fraction of EMT6/Ro cells to ~0·5. However, no cytotoxicity was observed in the EMT6/St/lu tumour after prolonged exposure to low MISO levels (achieved by nephrectomy) in the mouse (Brown & Yu, 1979) and, as can be seen in Figs 1, 3 & 4, no effect of multiple MISO alone was seen in the RIF-1 tumour in the present study. It seems unlikely, therefore, that enhancement by MISO is simply an additive toxicity.

It has been shown that preincubation of mammalian cells with MISO under hypoxic conditions sensitizes these cells to subsequent exposure to alkylating agents (Stratford *et al.*, 1980). Our data suggest similarities between the "preincubation effect" and the *in vivo* enhancement of cytotoxicity. In each case, cells must be exposed to MISO before the alkylating agent to show enhancement. However, hypoxia is a requirement for the "preincubation effect" (Stratford *et al.*, 1980), whereas our data show that different levels of hypoxia, which produce different responses to radiation (Brown *et al.*, 1980), do not affect chemosensitization (Figs 3 & 4). This, and the observation that normal tissues can be sensitized by large single doses of MISO (Law *et al.*, (1981), suggest that an *in vivo* manifestation of the preincubation effect is not the only mechanism involved.

While a fuller understanding of the process of MISO enhancement of alkylating-agent toxicity is highly desirable if the greatest clinical benefit is to be obtained with these combinations, our data give cause for optimism that, under currently acceptable conditions of administration, MISO can be combined with at least two widely used alkylating agents to achieve greater killing of tumour cells without a significant increase in normal-tissue complications.

The authors are grateful to Drs N. Y. Yu and W. Y. Koo, Ms R. Goddard, and J. L. Hazlehurst for their skilled assistance with these experiments. This investigation was funded by research grants CA-15201 and CA-25990 from the National Cancer Institute, D.H.E.W., U.S.A.

### REFERENCES

ADAMS, G. E. (1977) Hypoxic cell radiosensitizers for radiotherapy. In *Cancer: A Comprehensive Treatise*, Vol. 6, (Ed. Becker). New York: Plenum Press, p. 181.

ADAMS, G. E., MICHAEL, B. D., ASQUITH, J. C., SHENOY, M. A., WATTS, M. E. & WHILLANS, D. W. (1975) Rapid-mixing studies on the time-scale of radiation damage in cells. In *Radiation Research: Biomedical, Chemical and Physical Perspectives*, (Eds. Nygaard *et al.*). New York: Academic Press, p. 478.

BROWN, J. M. & YU, N. Y. (1979) Cytotoxicity of misonidazole *in vivo* under conditions of prolonged contact of drug with tumour cells. *Br. J. Radiol.*, **51**, 893.

BROWN, J. M., TWENTYMAN, P. R. & ZAMVIL, S. (1980) Response of the RIF-1 tumor *in vitro* and in C3H/Km mice to X-radiation (cell survival, regrowth delay and tumor control), chemotherapeutic agents and activated macrophages. *J. Natl. Cancer Inst.*, **64**, 605.

CLEMENT, J. J., GORMAN, M. S., WODINSKY, I., CATANE, R. & JOHNSON, R. K. (1980) Enhancement of antitumor activity of alkylating agents by the radiation sensitizer misonidazole. *Cancer Res.*, **40**, 4165.

CONROY, P. J., VON BURG, R. V., PASSALACQUA, W., PENNEY, D. P. & SUTHERLAND, R. M. (1979) Misonidazole neurotoxicity in the mouse: Evaluation of functional, pharmacokinetic, electrophysiologic and morphologic parameters. *Int. J. Radiat. Oncol. Biol. Phys.*, **5**, 983.

CONROY, P. J., SUTHERLAND, R. M. & PASSALACQUA, W. (1980) Misonidazole cytotoxicity *in vivo*: A comparison of large single doses with smaller doses and extended contact of the drug with tumor cells. *Radiat. Res.*, **83**, 169.

LAW, M. P., HIRST, D. G. & BROWN, J. M. (1981) The enhancing effect of misonidazole on the response of the RIF-1 tumour to cyclophosphamide. *Br. J. Cancer*, **44**, 208.

MARTIN, W. M. C., MCNALLY, N. J. & DERONDE, J. (1981) The potentiation of cyclophosphamide cytotoxicity by misonidazole. *Br. J. Cancer*, **43**, 756.

MOORE, B. A., PALCIC, B. & SKARSGARD, L. D. (1976) Radiosensitizing and toxic effects of the 2-nitroimidazole Ro-07-0582 in hypoxic mammalian cells. *Radiat. Res.*, **67**, 459.

MULCAHY, R. T., SIEMANN, D. W. & SUTHERLAND R. M. (1981) *In vivo* response of KHT sarcomas to combined chemotherapy with misonidazole and BCNU. *Br. J. Cancer*, **43**, 93.

ROSE, C. M., MILLAR, J. L., PEACOCK, J. H., PHELPS, T. A. & STEPHENS, T. (1980) Differential enhancement of melphalan cytotoxicity in tumor and normal tissue by misonidazole. In *Radiation Sensitizers: Their Use in the Clinical Management of Cancer*, (Ed. Brady). New York: Manor Publishing, p. 405.

SIEMANN, D. W. (1981) The *in vivo* combination of the nitroimidazole misonidazole and the chemotherapeutic agent CCNU. *Br. J. Cancer*, **43**, 367.

STRATFORD, I. J., ADAMS, G. E., HORSMAN, M. R. & 4 others (1980) The interaction of misonidazole with radiation, chemotherapeutic agents or heat: A preliminary report. *Cancer Clin. Trials*, **3**, 231.

TANNOCK, I. F. (1980) The *in vivo* interaction of anticancer drugs with misonidazole or metronidazole: Cyclophosphamide and BCNU. *Br. J. Cancer*, **42**, 871

TILL, J. E. & MCCULLOCH, E. A. (1961) A direct measurement of the radiation sensitivity of normal mouse bone marrow cells. *Radiat. Res.*, **14**, 213.

TWENTYMAN, P. R., KALLMAN, R. F. & BROWN, J. M. (1979) The effect of time between X-irradiation and chemotherapy on the growth of three solid mouse tumors. I. Adriamycin. *Int. J. Radiat. Oncol. Biol. Phys.*, **5**, 1255.

TWENTYMAN, P. R., BROWN, J. M., GRAY, J. W., FRANKO, A. J., SCOLES, M. A. & KALLMAN, R. F.

(1980) A new mouse tumor model system (RIF-1) for comparison of end point studies. **64,** 595.

TWENTYMAN, P. R. (1981) Modification of tumour and host response to cyclophosphamide by misonidazole and WR-2721. *Br. J. Cancer*, **43,** 745.

URTASUN, R. C., BAND, P., CHAPMAN, J. D., RABIN, H. R., WILSON, A. F. & FRYER, C. G. (1977) Clinical phase 1 study of the hypoxic cell radiosensitizer Ro 07-0582, a 2-nitroimidazole derivative. *Radiology*, **122,** 801.

WORKMAN, P., LITTLE, C. J., MARTEN, T. R. & 4 others (1978) Estimation of the hypoxic cell sensitizer misonidazole and its 0-demethylated metabolite in biological materials by reversed-phase liquid chromatography. *J. Chromatogr.*, **145,** 507.


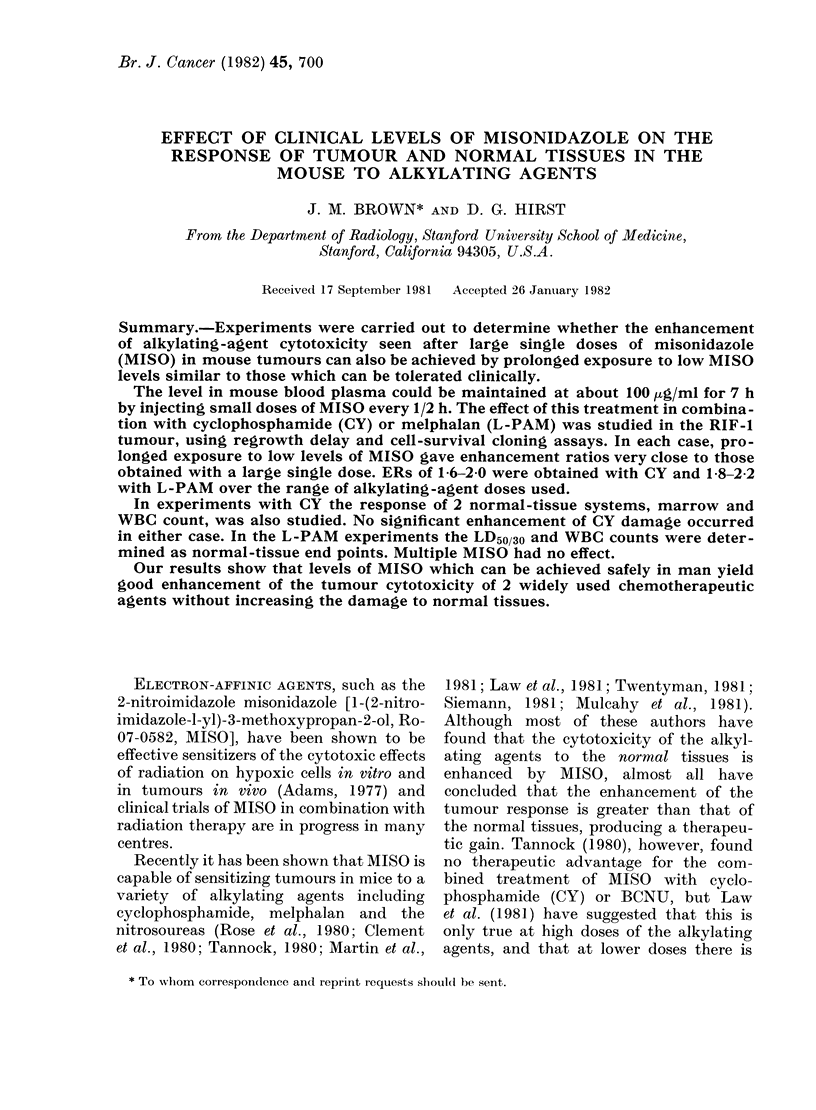

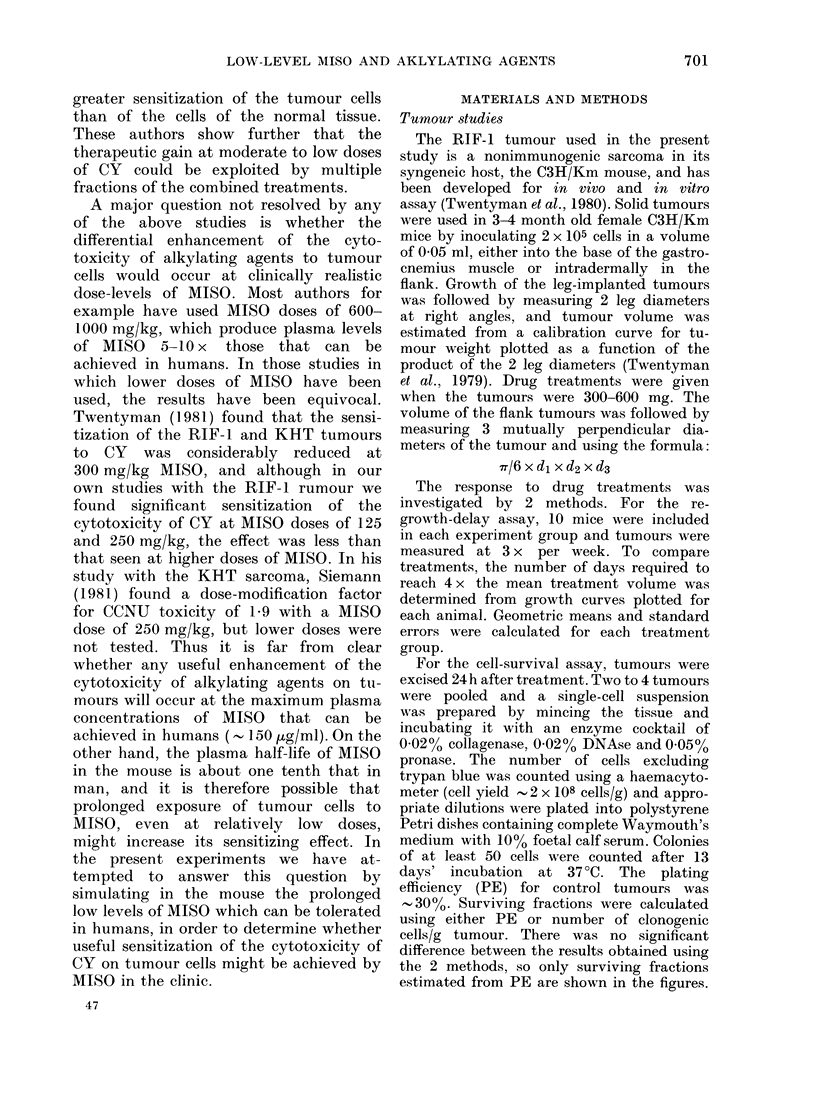

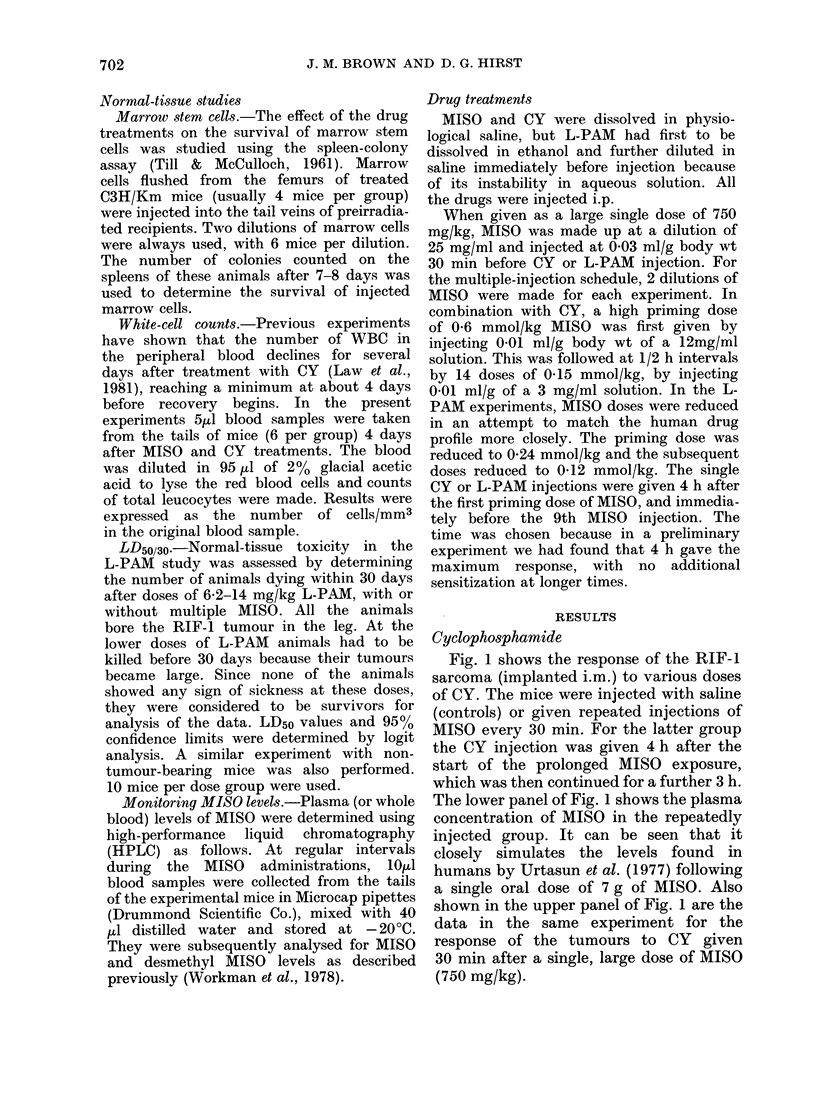

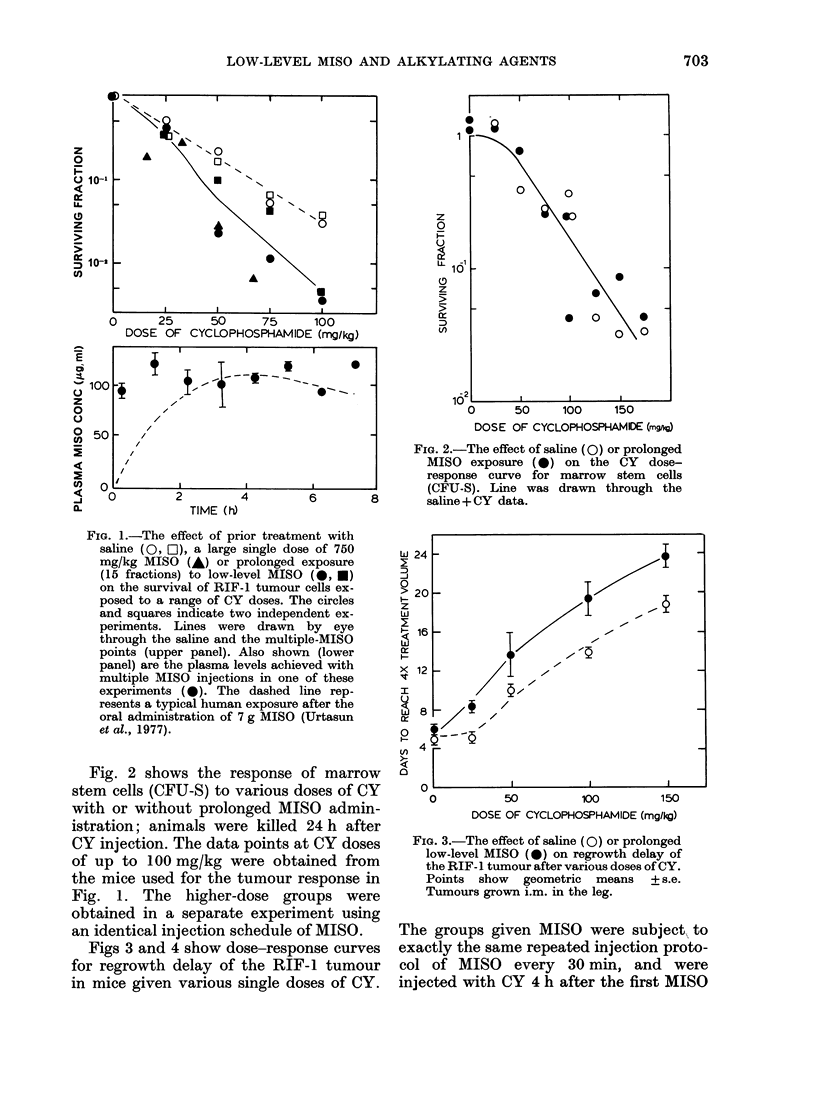

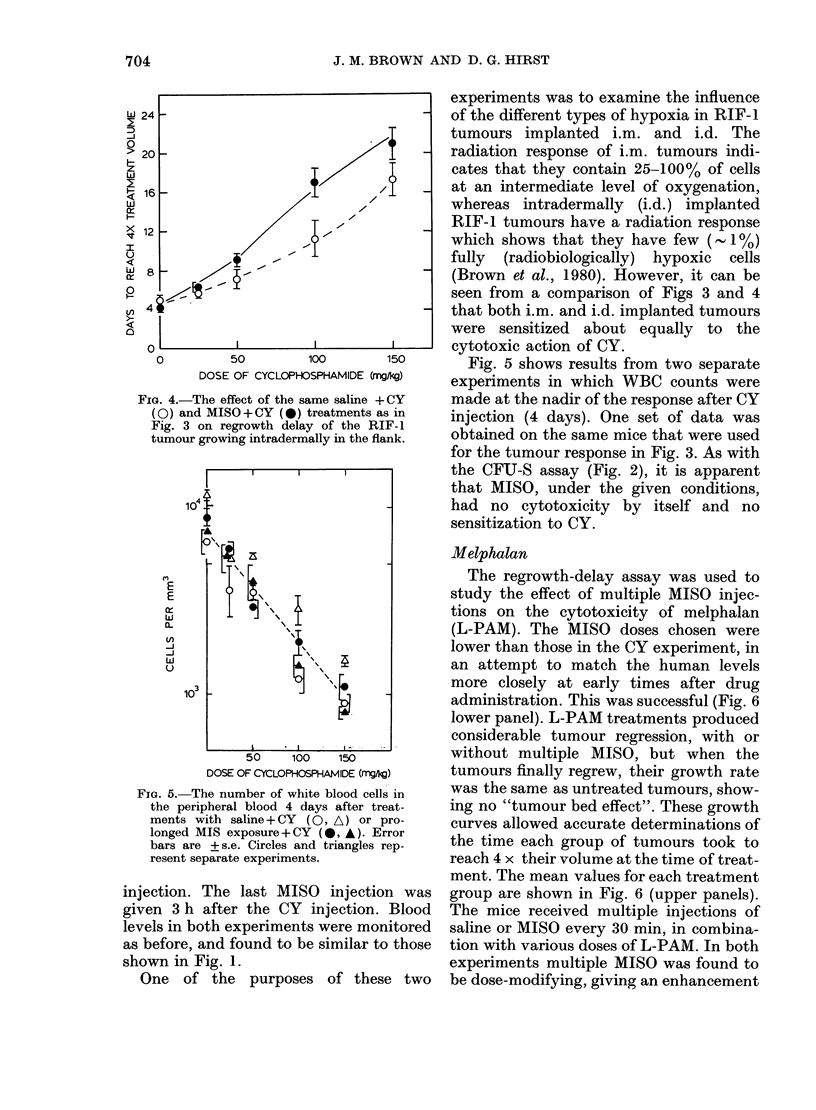

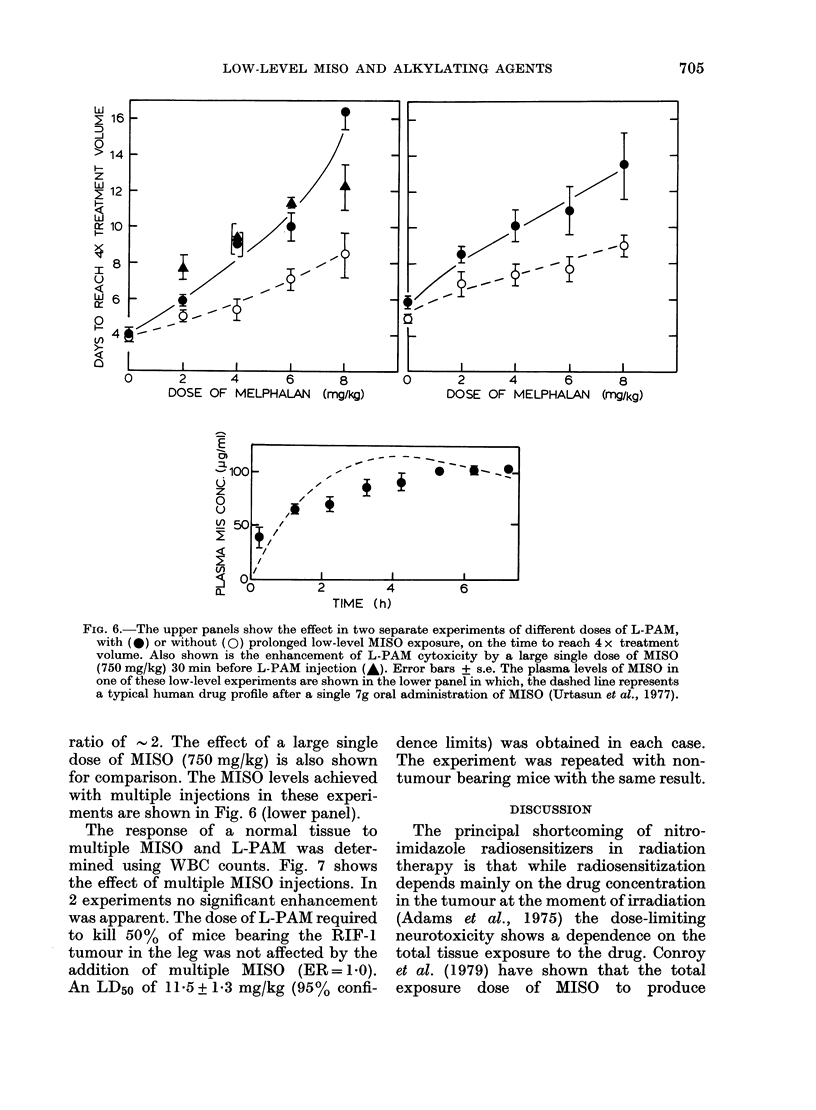

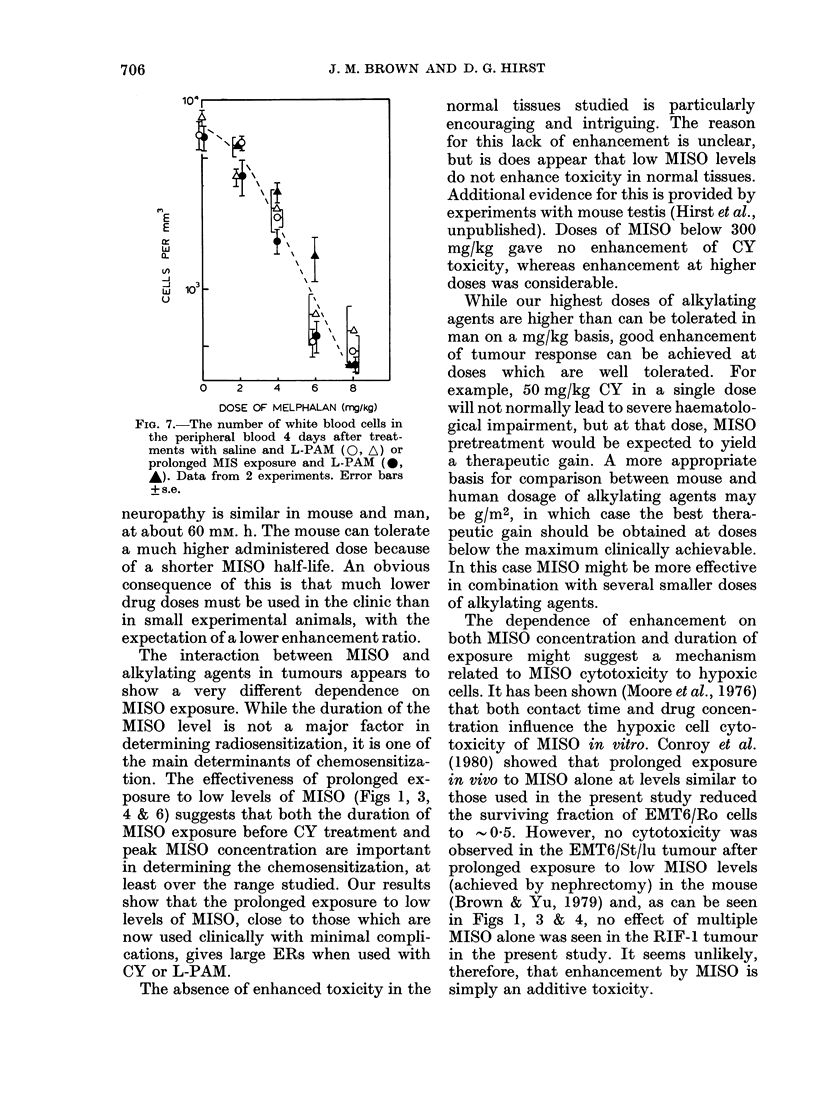

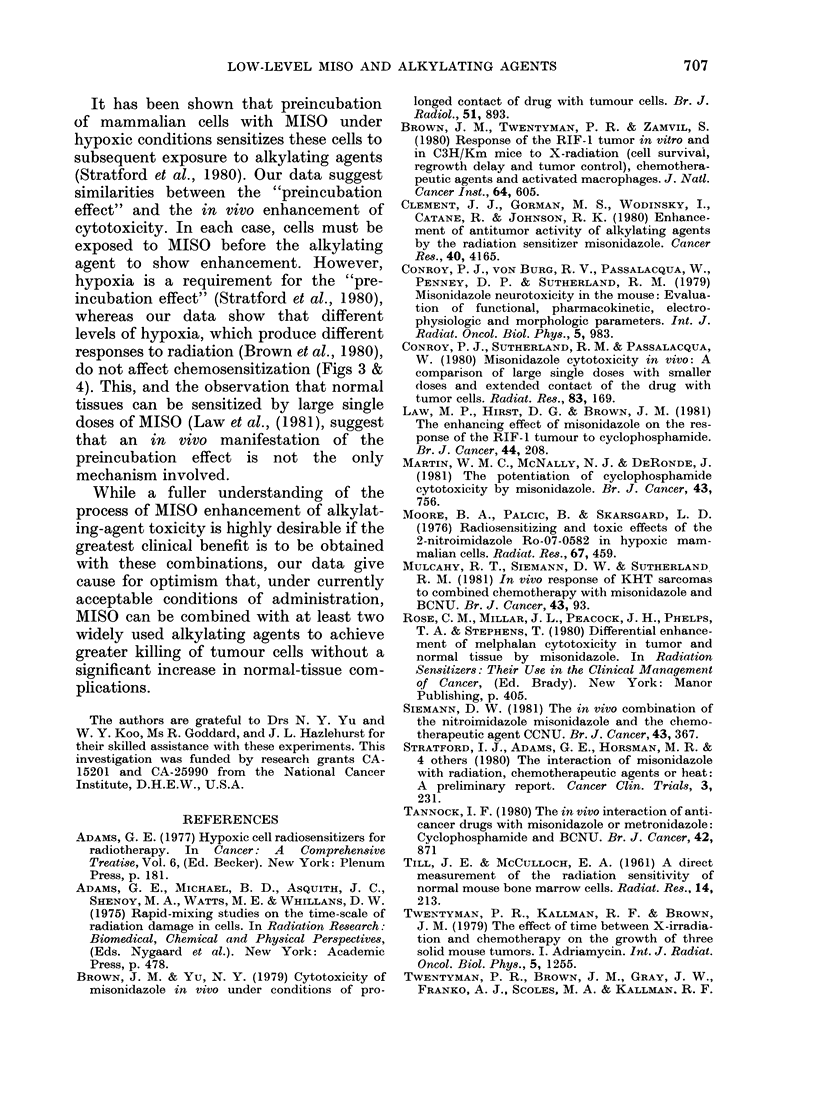

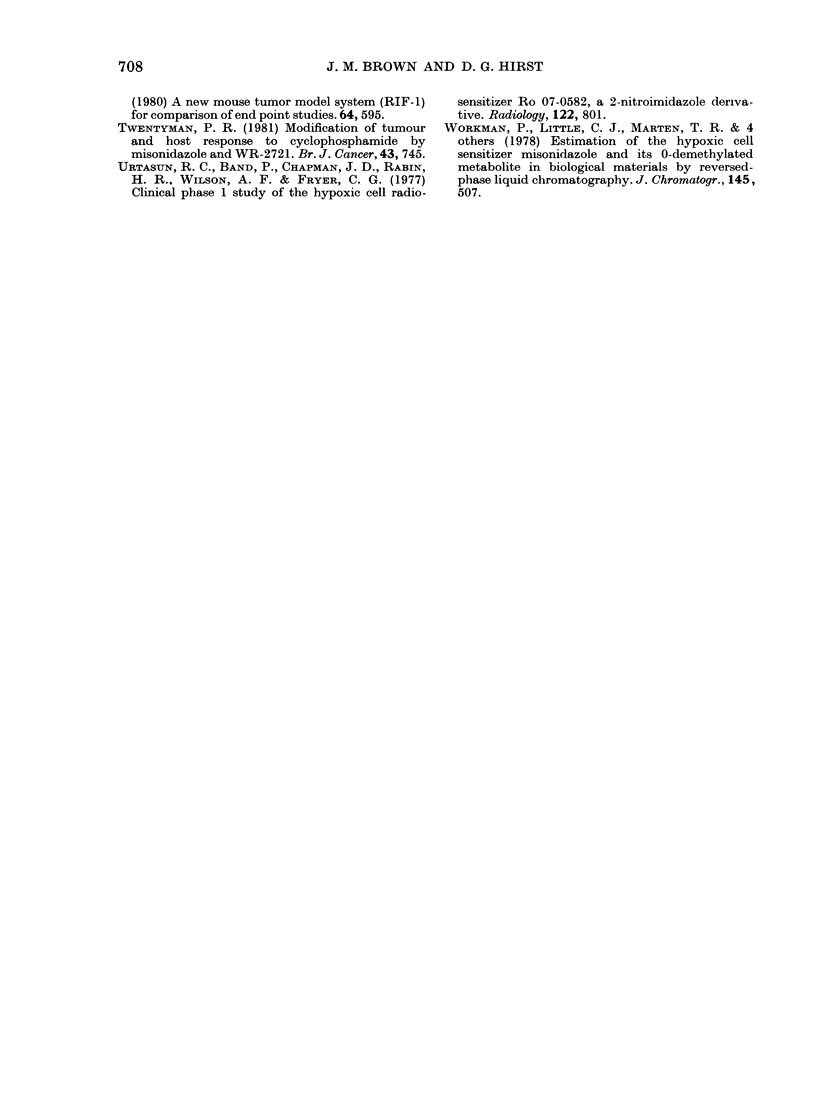


## References

[OCR_00907] Brown J. M., Twentyman P. R., Zamvil S. S. (1980). Response to the RIF-1 tumor in vitro and in C3H/Km mice to X-radiation (cell survival, regrowth delay, and tumor control), chemotherapeutic agents, and activated macrophages.. J Natl Cancer Inst.

[OCR_00900] Brown J. M., Yu N. Y. (1979). Cytotoxicity of misonidazole in vivo under conditions of prolonged contact of drug with the tumour cells.. Br J Radiol.

[OCR_00915] Clement J. J., Gorman M. S., Wodinsky I., Catane R., Johnson R. K. (1980). Enhancement of antitumor activity of alkylating agents by the radiation sensitizer misonidazole.. Cancer Res.

[OCR_00930] Conroy P. J., Sutherland R. M., Passalacqua W. (1980). Misonidazole cytotoxicity in vivo: a comparison of large single doses with smaller doses and extended contact of the drug with tumor cells.. Radiat Res.

[OCR_00922] Conroy P. J., Von Burg R., Passalacqua W., Penney D. P., Sutherland R. M. (1979). Misonidazole neurotoxicity in the mouse: evaluation of functional, pharmacokinetic, electrophysiologic and morphologic parameters.. Int J Radiat Oncol Biol Phys.

[OCR_00937] Law M. P., Hirst D. G., Brown J. M. (1981). Enhancing effect of misonidazole on the response of the RIF-1 tumour to cyclophosphamide.. Br J Cancer.

[OCR_00943] Martin W. M., McNally N. J., De Ronde J. (1981). Enhancement of the effect of cytotoxic drugs by radiosensitizers.. Br J Cancer.

[OCR_00949] Moore B. A., Palcic B., Skarsgard L. D. (1976). Radiosensitizing and toxic effects on the 2-nitroimidazole Ro-07-0582 in hypoxic mammation cells.. Radiat Res.

[OCR_00970] Siemann D. W. (1981). In vivo combination of misonidazole and the chemotherapeutic agent CCNU.. Br J Cancer.

[OCR_00975] Stratford I. J., Adams G. E., Horsman M. R., Kandaiya S., Rajaratnam S., Smith E., Williamson C. (1980). The interaction of misonidazole with radiation, chemotherapeutic agents, or heat: a preliminary report.. Cancer Clin Trials.

[OCR_00988] TILL J. E., McCULLOCH E. A. (1961). A direct measurement of the radiation sensitivity of normal mouse bone marrow cells.. Radiat Res.

[OCR_00982] Tannock I. F. (1980). In vivo interaction of anti-cancer drugs with misonidazole or metronidazole: cyclophosphamide and BCNU.. Br J Cancer.

[OCR_00994] Twentyman P. R., Kallman R. F., Brown J. M. (1979). The effect of time between X-irradiation and chemotherapy on the growth of three solid mouse tumors--I. Adriamycin.. Int J Radiat Oncol Biol Phys.

[OCR_01010] Twentyman P. R. (1981). Modification of tumour and host response to cyclophosphamide by misonidazole and by WR 2721.. Br J Cancer.

[OCR_01014] Urtasun R. C., Band P., Chapman J. D., Rabin H. R., Wilson A. F., Fryer C. G. (1977). Clinical phase I study of the hypoxic cell radiosensitizer RO-07-0582, a 2-nitroimidazole derivative.. Radiology.

[OCR_01022] Workman P., Little C. J., Marten T. R., Dale A. D., Ruane R. J., Flockhart I. R., Bleehen N. M. (1978). Estimation of the hypoxic cell-sensitiser misonidazole and its O-demethylated metabolite in biological materials by reversed-phase high-performance liquid chromatography.. J Chromatogr.

